# Predicting Seasonal Influenza Based on SARIMA Model, in Mainland China from 2005 to 2018

**DOI:** 10.3390/ijerph16234760

**Published:** 2019-11-27

**Authors:** Jing Cong, Mengmeng Ren, Shuyang Xie, Pingyu Wang

**Affiliations:** 1 Department of Epidemiology, Binzhou Medical University, YanTai 264003, China; cjboy123@163.com (J.C.); 17853589692@163.com (M.R.); 2 Department of Biochemistry and Molecular Biology, Binzhou Medical University, YanTai 264003, China; shuyangxie@aliyun.com

**Keywords:** SARIMA model, influenza, prediction

## Abstract

Seasonal influenza is one of the mandatorily monitored infectious diseases, in China. Making full use of the influenza surveillance data helps to predict seasonal influenza. In this study, a seasonal autoregressive integrated moving average (SARIMA) model was used to predict the influenza changes by analyzing monthly data of influenza incidence from January 2005 to December 2018, in China. The inter-annual incidence rate fluctuated from 2.76 to 55.07 per 100,000 individuals. The SARIMA (1, 0, 0) × (0, 1, 1) 12 model predicted that the influenza incidence in 2018 was similar to that of previous years, and it fitted the seasonal fluctuation. The relative errors between actual values and predicted values fluctuated from 0.0010 to 0.0137, which indicated that the predicted values matched the actual values well. This study demonstrated that the SARIMA model could effectively make short-term predictions of seasonal influenza.

## 1. Introduction

Influenza remains an increasing public health problem worldwide, especially since the 1918 influenza pandemic. Although many efforts have focused on measures and strategies to prevent and control influenza [1,2,3,4], it still results in significant mortality, health care capacity, and economic costs to society annually. A modeling study estimated that 291,243 to 645,832 seasonal influenza-associated respiratory deaths (4.0 to 8.8 per 100,000 individuals) occurred annually worldwide from 1999 to 2015 [5]. It estimated that 11.5% of lower respiratory tract infection episodes were attributable to influenza [6]. The expected annual losses from pandemic risk are about 0.6% of the global income [7]. The burden caused by influenza is higher in seasons dominated by A (H3N2) or A (H1N1) pdm 2009 influenza viruses but is lower in seasons where pre-pandemic A (H1N1) or influenza B accounts for the majority of cases [8]. In 2019, the WHO and partners launched the Global Influenza Strategy for 2019 to 2030 to strengthen seasonal prevention and control of future pandemics [9]. This strategy includes improving the influenza model and forecast. Therefore, it is important to use influenza surveillance data to model and forecast the influenza pandemics.

Time series analysis of infection data from analysis of specific models can improve the prevention system and forecast future values based on the previously observed values [10,11]. These models include seasonal autoregressive integrated moving average (SARIMA), neural network model, exponential smoothing, grey swing model, etc. [12,13,14,15]. The SARIMA model takes both overall trends and seasonal changes into account, which is widely used in modeling time series [13,14,15]. 

Our previous study demonstrated that there was a trend of increasing influenza incidence from 2005 to 2015, in mainland China [16]. China has a population of about 1.3 billion people, and the disease burden caused by influenza is still heavy. The mortality caused by influenza is around 8% of all respiratory deaths in China [17]. Considering that seasonal influenza is one of the notifiable infectious diseases in China, it is helpful to model and forecast influenza by analyzing the influenza surveillance data. Furthermore, the country-specific estimate should be updated periodically, and a certain model should be developed. Therefore, the SARIMA model was performed to analyze the changes of influenza from recent surveillance data of influenza from January 2005 to December 2018, in China. 

## 2. Materials

### 2.1. Date Collection

The total number of influenza cases and the monthly data of influenza incidence from January 2005 to December 2018 was provided by the website of the National Health Commission of the People’s Republic of China [18]. 

The Chinese government has established a web-based national notifiable infectious disease surveillance system for 39 infectious diseases, including influenza since 2003. Clinicians complete a standard case report card when they identify any probable, clinical, or laboratory-confirmed case of seasonal influenza-A and influenza-B. Then, the local epidemiologists do a field investigation when they receive the disease card using a standardized form, which improves the data accuracy [16,19]. 

### 2.2. SARIMA Model 

**Figure 1:**
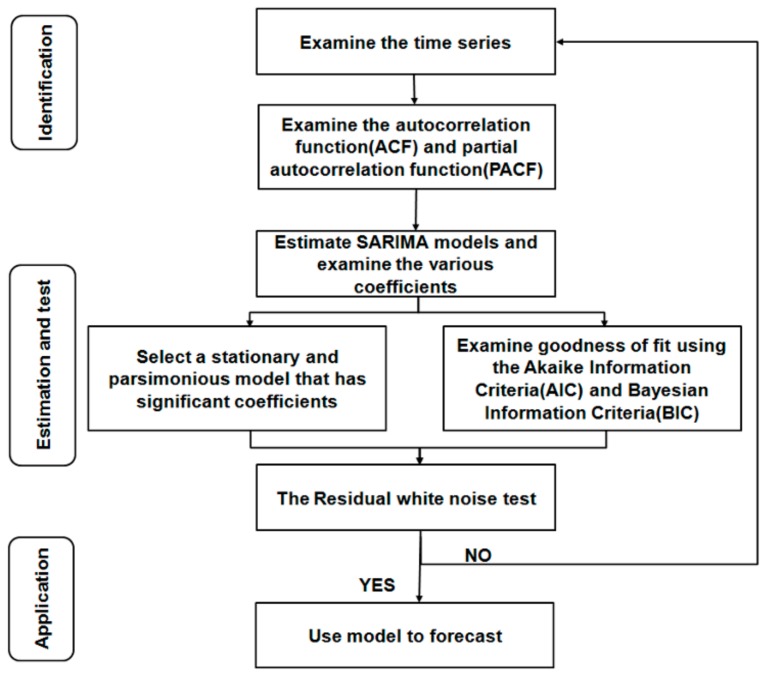
The process and method of seasonal autoregressive integrated moving average (SARIMA) model.

The data of influenza incidence from January 2005 to June 2018 was used as a training dataset, and that data from July 2018 to December 2018 was used as the forecasting dataset. We established and selected the best SARIMA model (p, d, q) × (P, D, Q) according to the steps introduced by Box and Jenkins [20] (Figure 1). Autoregressive lags, moving average lags, seasonal autoregressive lags, and seasonal moving average lags are indicated by p, q, P, and Q, respectively.

### 2.3. Statistical Analysis 

STATA 15.0 (Stata Corp., College Station, TX, USA) and SPSS 22.0 (SPSS Inc., Chicago, IL, USA) were performed to create the SARIMA model. 

## 3. Results

### 3.1. General Trend of Influenza Incidence 

**Figure 2:**
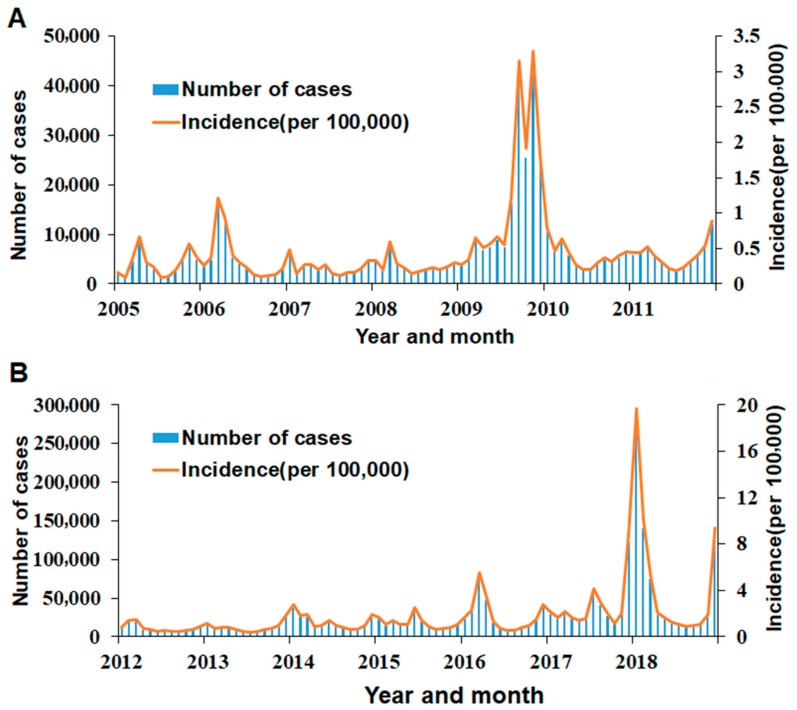
The influenza incidence in mainland China from 2005 to 2018: (**A**) Influenza cases and incidence from January 2005 to December 2011 and (**B**) influenza cases and incidence from January 2012 to December 2018.

A total of 2,686,180 influenza cases were reported in mainland China from January 2005 to December 2018. The annual incidence rate fluctuated from 2.76 to 55.07 per 100,000 individuals. Influenza occurred throughout the year, with two peaks in winter and spring (Figure 2). 

### 3.2. SARIMA Model 

Using the raw training data from January 2005 to June 2018, trend difference (d = 0) and seasonal difference (D = 1) were completed. The augmented Dickey−Fuller method was used to determine whether the sequence was stationary, and the result supported that the data was a stationary time series (t = −9.247, *p* < 0.001). 

**Figure 3:**
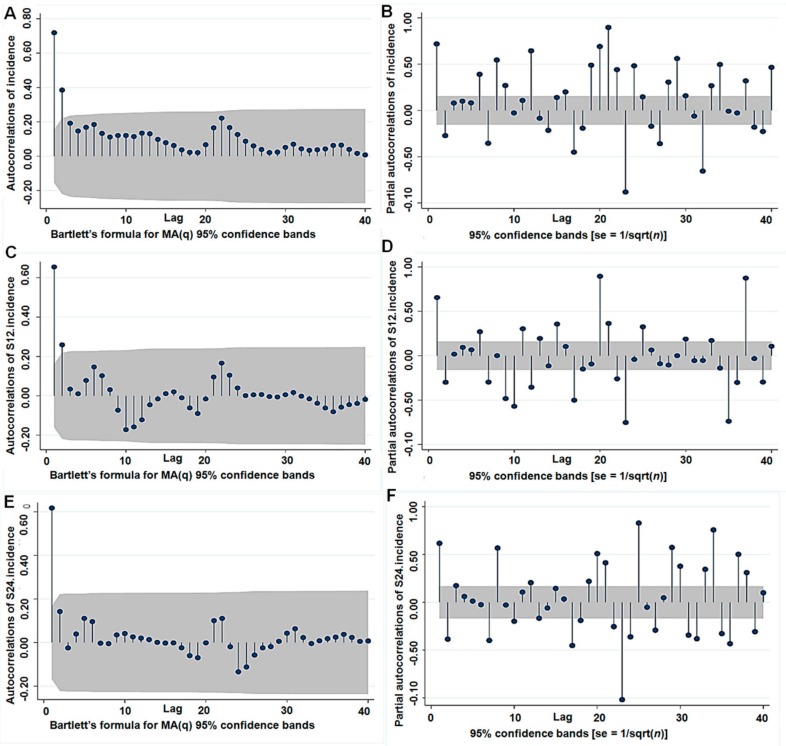
The ACF and PACF graphs for estimating the parameter: (**A**) The ACF graph of the raw data (d = 0 and D = 0), (**B**) the PACF graph of the raw data (d = 0 and D = 0), (**C**) the ACF graph of one-order seasonal difference data (d = 0 and D = 1), (**D**) the PACF graph of one-order seasonal difference data (d = 0 and D = 1), (**E**) the ACF graph of two-order seasonal difference data (d = 0 and D = 2), and (**F**) the PACF graph of two-order seasonal difference data (d = 0 and D = 2).

The auto-correlation Function (ACF) and partial correlation function (PACF) graphs were used to estimate the parameter ranges of p, P and q, Q (Figure 3). The ACF graph of one-order seasonal difference data (Figure 3C) and the PACF graph of one-order seasonal difference data (Figure 3D) showed better than the others. Then, some candidate SARIMA models were assessed to forecast future values based on the previously observed values (Table 1). On the basis of the results of the goodness-of-fit test statistics, SARIMA (1, 0, 0) × (0, 1, 1) 12 model was found to the optimal model, which had the lowest Akaike information criterion (AIC = 535.2955) and Bayesian information criterion (BIC = 544.3274). This model also passed the Ljung–Box Q Test (z = 25.607, *p* = 0.060). All the parameter estimates were significant (Table 1). 

**Figure 4:**
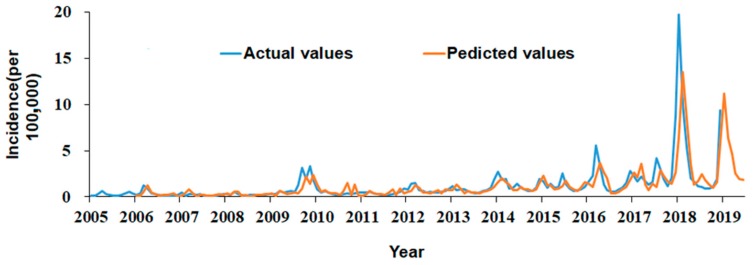
Comparison of actual and predicted incidence of influenza in mainland China.

The model forecasting effect was tested by comparing predicted values with the actual values. The results showed SARIMA (1, 0, 0) × (0, 1, 1) 12 model fitted the seasonal fluctuation well (Figure 4). Then, the model was used to forecast the influenza incidence from July to December 2018. The relative errors between actual values and predicted values fluctuated from 0.0010 to 0.0137. All actual values are among 95% CI of predicted values (Table 2). 

## 4. Discussion

Seasonal influenza is an acute respiratory infection caused by influenza viruses. Influenza surveillance to improve the influenza is the basis of influenza prevention and control. Global influenza surveillance has been conducted through the WHO’s Global Influenza Surveillance and Response System (GISRS) since 1952. The GISRS Network remains alert to timely recognize potential threats and minimize the impact of influenza epidemics and pandemics [21].

In many countries, more attention has been paid to influenza surveillance. The surveillance data types are usually used to establish a variety of influenza surveillance systems, including influenza-likely illness (ILI), acute respiratory infection (ARI), influenza cases, laboratory-confirmed influenza, Google flu trends, protein sequences, etc. [5,10,22,23,24,25]. In this study, the current influenza surveillance system covers the following contents: (i) ILI, (ii) ARI, (iii) outbreak surveillance, and (iv) notifiable infectious disease surveillance. We used reportable contagious disease surveillance data to analyze the trend of influenza, and our results demonstrated that seasonal influenza is one of the web-based national notifiable infectious diseases in China, since 2003. The surveillance data of this study are of high accuracy by quality control, which ensures the authenticity of these results. Since 2003, the Chinese government has improved the surveillance system. When clinicians identify any case of seasonal influenza-A and influenza-B, since 2003, they report it through the web-based national notifiable infectious disease surveillance system within 24 hours. Epidemiologists evaluate the report rigorously, which is helpful to reduce the surveillance bias and enhance the data accuracy.

Influenza is affected by many biological, behavioral, and environmental factors, which lead to a seasonal variation. Previous studies have assumed that influenza is an annual spring or winter epidemic in some cities of China [26,27]. The data from the 14-year surveillance in this study are in agreement that influenza has seasonal variation in winter or spring. Seasonality exists with two peaks in winter and spring, consistent with well-documented peaks for influenza-A and influenza-B [25,26]. The reasons for seasonal epidemics may be related to factors such as a vast population, high residential density, and crowded living conditions, the variability of influenza viruses, diversity of geography, cold winter weather, low vaccination rate, etc. [27,28,29].

The multiple factors cause difficulties in modeling the influenza pandemic. Several approaches are applied to make these models. These approaches can be categorized as follows: time series models, compartmental modes, agent-based models, met population models, and approaches in meteorology. Time series analysis has the advantage of forecasting the incidence without focusing on specific risk factors. It uses the number of patients in the past as features to forecast the number of patients in the future as the response. The SARIMA model is performed over a time series in an automated fashion to maximize prediction accuracy. In addition, it takes both overall trends and seasonal changes into account and has been widely used for time series analysis. The SARIMA model typically assumes that future values in about three to six months can be predicted based on previously observed values [29]. Accordingly, we constructed the SARIMA (1, 0, 0) × (0, 1, 1) _12_ model to forecast influenza incidence. This model forecasted that the influenza incidence from July 2018 to December 2018 was similar to that of previous years, and there was also a seasonal variation during winter. Our results demonstrated that the predicted values matched the actual values well, supporting that the SARIMA model is effective in the prevention and control of influenza. It can capture trends and periodic changes. 

## 5. Limitations

Several limitations should be noted in this study. First, only the SARIMA model was used and we assumed that there was a linear relationship between influenza incidence and its factors, such as exposure, susceptibility, access to care, etc. Many environmental and natural factors are dynamic, so the parameters of the SARIMA model should be periodically reassessed according to continuously updated data. Second, the surveillance data of this study cannot exclude surveillance bias in spite of quality control, which may affect our results to some extent. Third, we only used the data of all mainland China for prediction, analysis of subgroups (the South of China and the North of China) could be more reasonable. Fourth, we collected only monthly data, and weekly reporting could have better accuracy.

## 6. Conclusions

This work demonstrates that influenza occurred throughout the year with two peaks in winter and spring, in mainland China, which reminds us that influenza never goes away. Additional practical efforts should focus on reducing the burden of seasonal influenza. Our results also indicate that the SARIMA model can make short-term predictions of seasonal influenza effectively, and it is helpful to decision makers to allocate public health resources. 

## Figures and Tables

**Table 1:** Comparison of candidate SARIMA models.

Model	Estimate	Z	*p*-Value	Ljung-Box Q Test			AIC	BIC	RMSE	MAPE
				Statistics	DF	*p*-Value				
SARIMA (0,0,1) (0,1,1)_12_	-	-	-	22.753	16	0.121	541.661	550.692	1.439	48.744
q	0.654	11.00	0.000	-	-	-	-	-	-	-
Q	−0.415	−2.17	0.030	-	-	-	-	-	-	-
SARIMA (1,0,0) (0,1,1)_12_	-	-	-	25.607	16	0.060	535.296	544.327	1.407	44.280
p	0.668	25.68	0.000	-	-	-	-	-	-	-
Q	−0.445	−2.24	0.025	-	-	-	-	-	-	-
SARIMA (1,0,1) (0,1,1)_12_	-	-	-	8.157	15	0.917	523.172	535.214	1.345	44.137
p	0.481	3.15	0.002	-	-	-	-	-	-	-
q	−0.393	−3.772	0.074	-	-	-	-	-	-	-
Q	0.473	−2.53	0.012	-	-	-	-	-	-	-
SARIMA (1,0,1) (1,1,1)_12_	-	-	-	7.916	14	0.894	525.083	540.136	1.348	44.021
p	0.476	2.93	0.003	-	-	-	-	-	-	-
q	0.399	1.76	0.078	-	-	-	-	-	-	-
P	−0.080	−0.10	0.923	-	-	-	-	-	-	-
Q	−0.425	−0.50	0.615	-	-	-	-	-	-	-

AIC: Akaike information criterion; BIC: Bayesian information criterion; RMSE: root mean squared error; MAPE: mean absolute percent error; DF: degree of freedom.

**Table 2:** Comparison of predicted values and actual values form July to December 2018 (per 100,000 population).

Month	Actual Value	Predicted Value	Relative Error	95%CI	
				LCL	UCL
July	1.04	2.47	0.0137	−0.49	4.82
August	0.88	1.82	0.0106	−1.83	5.22
September	0.95	1.34	0.0042	−2.4	4.99
October	1.06	0.96	0.0010	−2.79	4.69
November	1.93	1.61	0.0017	−2.15	5.34
December	9.35	5.72	0.0039	1.86	9.36

LCL: lower confidence limit; UCL: upper confidence limit.
